# Olfactory dysfunction in patients with multiple sclerosis; A systematic review and meta-analysis

**DOI:** 10.1371/journal.pone.0266492

**Published:** 2022-04-19

**Authors:** Omid Mirmosayyeb, Narges Ebrahimi, Mahdi Barzegar, Alireza Afshari-Safavi, Sara Bagherieh, Vahid Shaygannejad

**Affiliations:** 1 Department of Neurology, School of Medicine, Isfahan University of Medical Sciences, Isfahan, Iran; 2 Isfahan Neurosciences Research Center, Isfahan University of Medical Sciences, Isfahan, Iran; 3 Department of Biostatistics and Epidemiology, Faculty of Health, North Khorasan University of Medical Sciences, Bojnurd, Iran; IRCCS San Raffaele Scientific Research Institute, ITALY

## Abstract

**Background:**

The importance and prevalence of olfactory dysfunction is recently gaining attention in patients with multiple sclerosis (MS) as a result of their chronic inflammatory disease, yet different prevalence rates are reported for it. Therefore, we have designed this systematic review to estimate the pooled prevalence of olfactory dysfunction in patients with MS. To our knowledge, this is the first systematic review and meta-analysis on the prevalence of olfactory dysfunction in MS patients.

**Methods:**

We searched PubMed, Scopus, EMBASE, Web of Science, ProQuest, and gray literature including references from the identified studies, review studies, and conference abstracts which were published up to January 2021. Articles that were relevant to our topic and could provide information regarding the prevalence of olfactory dysfunction, or the scores of smell threshold, discrimination, or identification (TDI) among MS patients and healthy individuals were included. The pooled prevalence was calculated using a random-effects model and a funnel plot and Egger’s regression test were used to see publication bias.

**Results:**

The literature search found 1630 articles. After eliminating duplicates, 897 articles remained. Two conference abstracts were included for final analysis. A total of 1099 MS cases and 299 MS patients with olfactory dysfunction were included in the analysis. The pooled prevalence of olfactory dysfunction in the included studies was 27.2%. Also, the overall TDI score in MS patients was lower than that in the control group, and the level of Threshold, Discrimination, and Identification per se were lower in MS compared with control respectively.

**Conclusion:**

The results of this systematic review show that the prevalence of olfactory dysfunction in MS patients is high and more attention needs to be drawn to this aspect of MS.

## Introduction

Olfaction is one of the most overlooked sensations of the human being. It is the means to the perception of smell, recognizing imminent dangers, and even storing memories and emotions [1]. The exact mechanism by which olfaction is mediated is still not completely clear to this date, so far, it has been revealed that an interaction between odor receptors and odor molecules initiate the process, leading to the production of olfactory signals which travel through the olfactory nerves to the Central Nervous System (CNS) [2]. This path is then followed by the storage of the smell as memories within the CNS for faster, more appropriate, and more reliable reactions in case of future encounters [3].

Multiple Sclerosis (MS) is an autoimmune disease, characterized by demyelination of CNS tissue. MS is a lifelong condition that can affect the brain and spinal cord, leading to a wide range of symptoms, including problems with vision, motor control, cognitive abilities, balance, and sensation. Olfaction is also affected in MS and olfaction impairment renders a lower quality of life in MS patients as olfaction ability is in direct contact with patients’ physical, behavioral, and cognitive state [2, 4, 5]. Olfaction is shown to be prone to impairment in three main aspects, including threshold, discrimination, and identification. Olfactory dysfunctions are reported as one the most common manifestations in the initial stages of certain CNS diseases, including Alzheimer’s disease and Parkinson’s disease [6]. Moreover, numerous studies have pointed out the presumable connection between olfactory disturbances and not only neurodegenerative diseases but also autoimmune ones, such as (MS) [7]. Several factors can contribute to the olfactory dysfunction MS patients occasionally report, some of which include persistent inflammation within the CNS, demyelination of olfactory bulbs, and the burden of plaque in brain areas associated with the olfactory system. Previous original articles have shown that the prevalence of olfactory disruption is higher among MS patients than healthy individuals [8].

Previous studies have provided conflicting evidence on determining the specific aspect of olfaction that suffers the most among MS patients, such aspects include Threshold, Discrimination, and Identification (TDI) dysfunction. They have also done so using different tools including but not limited to Sniffin’ Sticks test [9], the University of Pennsylvania Smell Identification Test (UPSIT) [10], the Brief Smell Identification Test (B-SIT) [11], and the Quick Smell Identification Test (Q-SIT) [12]. Such studies also lack coherence regarding the prevalence they report, with numbers ranging from 20% to 40% [13]. It is crucial to study olfactory dysfunction as it plays a major role in diminishing one’s quality of life [6], and also because there is growing evidence that the degree to which MS patients present with olfactory problems can be used as a potential prognostic factor [14]. On the other hand, the prevalence of olfactory disturbance among MS patients has been reported in various studies, among different sample sizes with different MS subtypes. Consequently, we designed this systematic review and meta-analysis to estimate the pooled prevalence of olfactory dysfunction among MS patients. The aims of this study are to: 1- Estimate the raw pooled prevalence of olfactory dysfunction among MS patients and 2- Take a step further and compare the TDI score among MS patients and healthy individuals.

## Methods

### Literature search

We conducted a systematic computerized search using five data banks: PubMed (MedLine), Scopus, web of science, and Embase (via Elsevier), and ProQuest. We also searched the gray literature including references from the identified studies, reviews studies, and conference abstracts which were published up to January 2021.

### Inclusion criteria

Studies reporting the prevalence of olfactory dysfunction or the scores of Thresholds, Discrimination, and Identification (TDI) among MS participants regardless of the diagnostic method with a sample size of over at least 10 patients were included.

Nevertheless, case reports and case series articles, articles that were written in any language other than English, and any published studies before 1990 were excluded due to the numerous differences in diagnostic approaches and clinical terms and definitions [15], and articles published after the end of 2020 were not included due to the timeline of our search which was covered studies up to January 2021.

### Data search and extraction

We conducted a systematic computerized search using five data banks: PubMed (Medline), Scopus, web of science, Embase, and ProQuest. We also searched the gray literature including references from the identified studies, reviews studies, and conference abstracts which were published up to January 2021.

We used Mesh terms and text words to generate a syntax that included two components.

“Olfaction Disorder,” “Smell Disorder”, “smell dysfunction”, “olfactory agnosia”, “agnosias for smell”, “dysfunction AND smell”, “olfactory impairment”, “impairment AND olfactory”, “sense of smell”, “smell sense”, “loss of smell”, “smell loss”, “Cacosmia”, “Dysosmia”, “Anosmia”, “paraosmia”, “hyposmia”, “agnosias”, and “agnosia AND olfactory” were the keywords we used to describe olfactory dysfunction; and also “multiple sclerosis”, “MS”, “disseminated sclerosis”, “Sclerosis AND multiple”, “sclerosis AND disseminated”, “acute fulminating”, and “acute fulminating” were the keywords we used to identify the other search component. Additionally, we customized our search syntax (query) for each data bank.

Two researchers (NE and SB) independently screened the articles. Any disagreement between the aforementioned researchers would be addressed by the senior researcher of the team (OM). The data extraction table included first author, region of study, date of publication, type of study, sample size of case and control group, and the demographic variables for case and control such as sex and mean of age. Other variables that we collected in our table included the exact name of the olfaction screening test, MS subtype, disease duration, EDSS score, number of hyposmia and anosmia in both case and control, plus the mean and standard deviation of the Threshold, Discrimination, and Identification (TDI) scores if applicable. Had any of the included articles used over one diagnostic method, each different methods would have been mentioned in a separate row of the table with its respective data. We used Systematic Review and Meta-Analysis statement (PRISMA) guideline. In case necessary data were missing from the eligible studies, emails were sent to the first and corresponding authors of the studies to retrieve any relevant data.

Furthermore, the olfaction diagnosis extraction form consisted of the total number of patients with olfactory dysfunction, hyposmia, anosmia, microsmia, and identification, threshold, and discrimination dysfunction.

All these variables were extracted from both MS and control group. In the present study, the control group represents healthy individuals without any neurologic disorders and/or diseases. The full list of the included studies is available in the reference section [1, 3, 6–8, 13, 14, 16–37].

### Statistical analysis

A statistical test for between study heterogeneity was performed by I-square (I^2^) and Cochran’s chi-square test. If evidence of heterogeneity was observed (I^2 > 50%), a random effect model was used. Forest plot was conducted to demonstrate the prevalence of olfactory in each study and the pooled estimate of prevalence with their 95% confidence intervals (95% CI). Due to the heterogeneity observed in the studies, a subgroup analysis was performed by the four major variables including sample size (≤ 50 and > 50), publication year (≤ 2010 and > 2010), EDSS (≤ 3 and > 3) and disease duration (≤ 10 and > 10) to figure out the source of the heterogeneity. The cut-offs used in the subgroups were determined based on the data provided in the included studies. As such, studies with sample sizes below 50 were considered to have reported a relatively small number of patients [6, 36], studies published prior to 2010 were more likely to have used different diagnostic methods [26, 38], patients with EDSS scores below 3 and disease durations below 10 years were considered to be less disabled to their MS disease [3, 31, 39]. Publication bias was assessed using funnel plot of logit transformed prevalence and Egger’s test. The Trim and fill approach were applied to obtain an adjusted effect size, when evidence of publication bias was observed. level of statistical significance was considered to be less than 0.05. All statistical analyses were done using Stata 14 software (Stata Corporation, College Station, Texas, USA).

## Results

**Fig 1:**
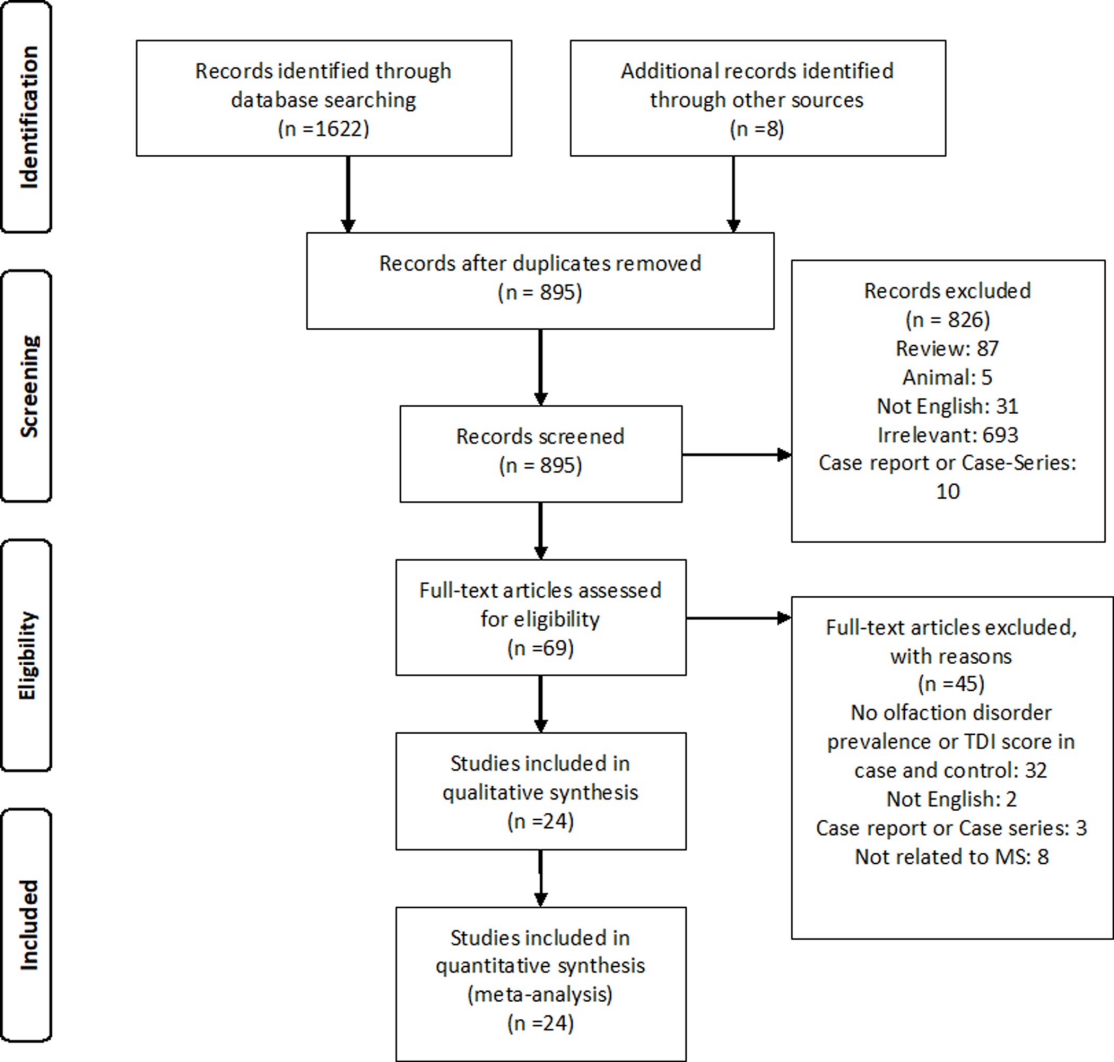
Flow diagram summarizing the selection of eligible studies.

The literature search found 1630 articles. After eliminating duplicates, 897 articles remained (Fig 1). Two conference abstracts were included for final analysis. A total of 1099 MS cases and 299 MS patients with olfactory dysfunction were included in the analysis. For those included articles that had used more than one diagnostic method, we assigned separate rows and a letter in parenthesis for each of their methods. Hence, some articles have been mentioned more than once in Table 1 pertaining to the article’s different diagnostic means. As such, Bsteh (a) and Bsteh(b), Hawkes (a) and Hawkes (b), Schmidt (a), Schmidt (b), Schmidt (c), and Schmidt (d), and Dahlsett (a) pertain to the different diagnostic methods that the aforementioned authors have used in their studies.

**Table 1:** Basic characteristics of the included studies.

Author	Study design	Country	MS sample	# MS Female/male	MS Age (years)	MS type	Disease Duration (years)	EDSS	#MS smokers	Control sample	Sample size of control group Female/Male	Olfactory diagnosis criteria
Okadaa, 2020	Cross section	Japan	40	F:32 M: 8	Med 38.5 Range 19–64	RRMS 40	Med 3.5 Range 1–24	Med: 1 Range 0–3	4	40	F:32 M:8	OSIT-J
Li-Min Li, 2018	Cross section	China	37	F: 20 M: 17	Mean: 42.5 SD: 12.6	NR	Mean: 5.3 SD: 5.1	Mean: 2.9 SD: 1.3	NR	-	-	Japanese T&T olfactometer test
Bsteh, 2018	Cross section	Austria	28	F: 20 M: 8	Mean: 34.6 SD: 8.4	RRMS 28	NR	Med: 2 Range 0–6.5	9	-	-	Sniffin sticks test
			27	F: 21 M: 6	Mean: 33.7 SD: 9	Stable MS	NR	Med: 1.5 Range 0–6.5	9	-	-	Sniffin sticks test
Carotenuto, 2018	Cross section	Italy	55	-	Mean: 45.9 SD: 14.25	RRMS 33 SPMS 22	Med 10.60 Range 5–41.68	Med: 4 Range 1.5–7.5	23	20	F:10 M:10	UPSIT
Uecker, 2017	Cohort	Germany	20	F: 15 M: 5	Mean: 44.9 SD: 10.10	RRMS 16 PPMS 4	3.2	Mean: 2 2	NR	-	-	TDI Test
Schmidt, 2017	Case control	Germany	14	NR	NR	PPMS 14	NR	NR	NR	14	NR	Tripartide TDI test
Schmidt, 2017	Case control	Germany	32	F:13 M: 19	Mean: 53.4 SD: 9.3	PPMS 32	Mean:11.3 SD: 8.4	Mean: 4.90 SD: 2.10	NR	32	F:17 M:15	Sniffin sticks test
			32	F:13 M:19	Mean: 35.5 SD: 9.3	RRMS 32	Mean:5.6 SD:5.9	Mean: 2.6 SD: 1.80				
Bsteh, 2017	Cohort	Switzerlan	141	F:112 M:29	NR	RRMS 128 SPMS 9 PPMS 4	NR	NR	43	30	F:22 M:8	Sniffin sticks test
Caglayan, 2016	Case control	Turkey	30	F:21 M:9	Mean: 34.3 SD: 9.8	RRMS 27 SPMS 3	Mean month: 47.7 SD month: 48	Mean: 1.91 SD: 1.57	10	30	F:21 M:9	Sniffin sticks test
Ekmekci, 2016	Case control	Turkey	30	-	NR	RRMS 15 SPMS 15	NR	NR	NR	20	NR	MediSense Taste Spray/Quick Smell Identification Test
Li-Min Li, 2015	Case control	China	26	F:15 M:11	Mean: 41.3 SD: 13.7	NR	Mean:5.2 SD:5.7	NR	NR	26	F:16 M:10	T&T olfactometer test kit
Holinski, 2014	Cross section	Germany	20	F: 13 M: 7	Mean: 39.5 SD: 11	RRMS 17 SPMS 1 PPMS 2	60.7 months 60.4 months	Mean: 3.10 SD: 1.60	NR	-	-	OEP
Caminiti, 2014	Case control	Italy	23	NR	NR	NR	NR	NR	NR	30	F:18 M:12	OERP
Dahlsett, 2012	Case control	Germany	30	F: 20 M: 10	Mean: 42.6 SD: 12.10	NR	Med: 4.2 Range: 1.3–11.8	Mean: 3.4 SD: 1.9	15	30	F: 20 M: 10	Sniffin sticks test
Silva, 2012	Cohort	Portugal	153	F: 107 M: 46	Mean: 41.91 SD: 11.28	RRMS 121 SPMS 16 PPMS 16	Mean:11.6 SD:8.50	Mean: 2.92 SD: 2.25	28	165	F:128 M:27	B-SIT
Lutterotti, 2011	Cross section	Italy	50	F: 35 M:15	Mean: 36.80 SD: 9.70	RRMS 37 SPMS 6 PPMS 2	Mean: 8.30 SD: 8.20	Med 2 Range 0–7	22	30	F:29 M:1	Sniffin sticks test
Goektas, 2011	Case control	Germany	36	F:25 M: 11	Mean: 41.5 SD: 12.20	RRMS 25 SPMS 4 PPMS 5	Mean:6.20 SD:7.80	Mean: 3.30 SD: 2.10	17	36	NR	Tripartide TDI test
Fleiner, 2010	Case control	Germany	16	F: 11 M: 5	Mean: 45.9 SD: 11.26	RRMS 8 SPMS 4 PPMS 4	Med: 7.17 Range: 3.56–13	Mean: 3.66 SD: 2.15	10	16	F:11 M:5	SSIT
Zorzon, 2000	Case control	Italy	40	F:25 M:15	Mean: 37.4 SD: 8.1	NR	NR	NR	NR	40	F:25 M:15	CC-SIT
Zivadinov, 1999	Case control	Italy	40	F 25 M 15	Mean: 37.4 SD: 8.10	RRMS 32 SPMS 2 PPMS 6	Mean:10.3 SD: 6.7	Mean: 2.5 SD: 1.8	15	40	F: 25 M: 15	CC-SIT
Hawkes, 1998	Case control	UK	72	F 43 M 29	43	NR	13	NR	NR	156	F: 99 M: 57	UPSIT OEP
Doty, 1998	Cross section	USA	26	F:17 M:9	42.11	NR	NR	NR	NR	-	-	UPSIT
Hawkes, 1997	Case control	UK	72	F: 43 M: 29	43.9	NR	11	NR	NR	96	F:57 M:39	UPSIT
			45	NR	NR	NR	NR	NR	NR	47	F:29 M:18	OEP
Lawrence, 1996	Case control	USA	16	F:11 M:5	40.1	NR	Mean: 12.3 SD:14.4	Mean: 5.20 SD: 2.70	NR	14	F:10 M:4	UPSIT

### Critical appraisal

**Fig 2:**
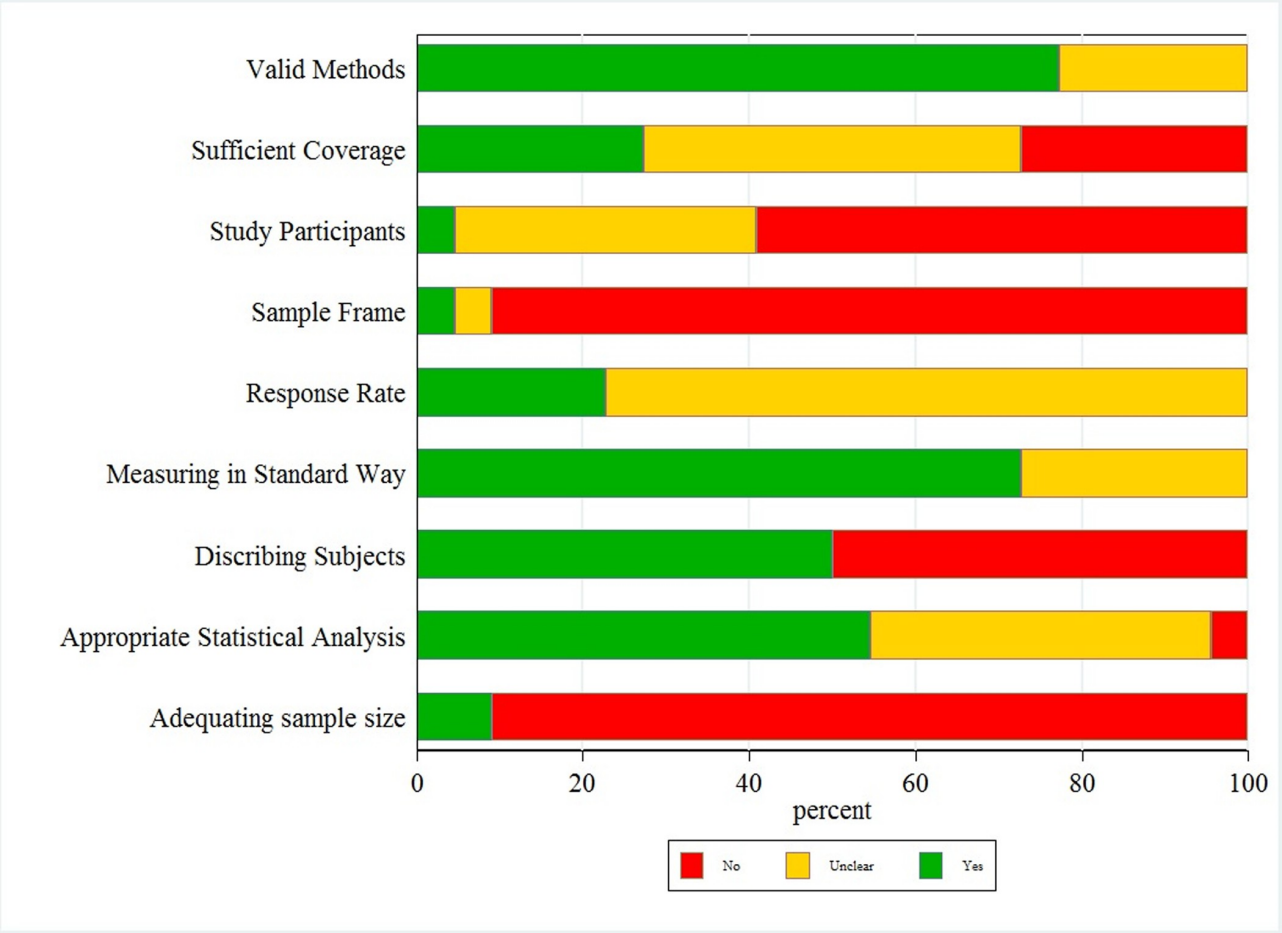
Quality assessment of the included studies based on the JBI checklist.

The quality of all the included articles was assessed using the Joanna Briggs Institute (JBI) critical appraisal checklist. The JBI checklist is the preferred tool for measuring the quality of descriptive studies reporting prevalence data and has a system of ranking articles based on the number of “YES” answers they earn according to its questions. The number of “YES” answers an article can earn ranges between 0 to 9 [40]. Using this checklist, 11 Of the included studies earned less than 4 “YES” answers, 10 studies earned between 4 to 6 “YES” answers, and 1 study earned more than 6 “YES” answers (*Fig 2*, *S1 Table, and S1 Fig*).

### Prevalence estimates

**Fig 3:**
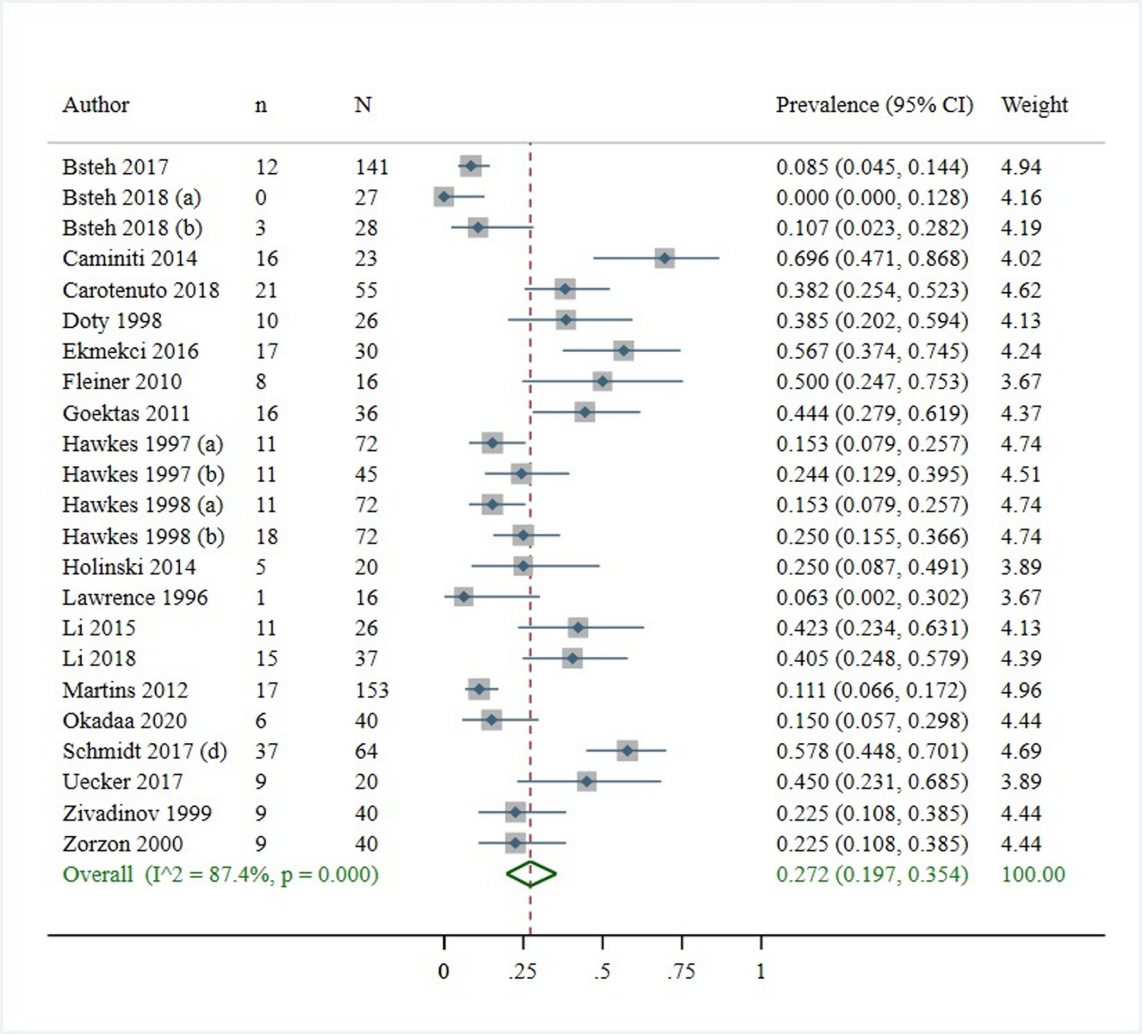
The pooled prevalence of olfactory among patients with MS.

**Fig 4:**
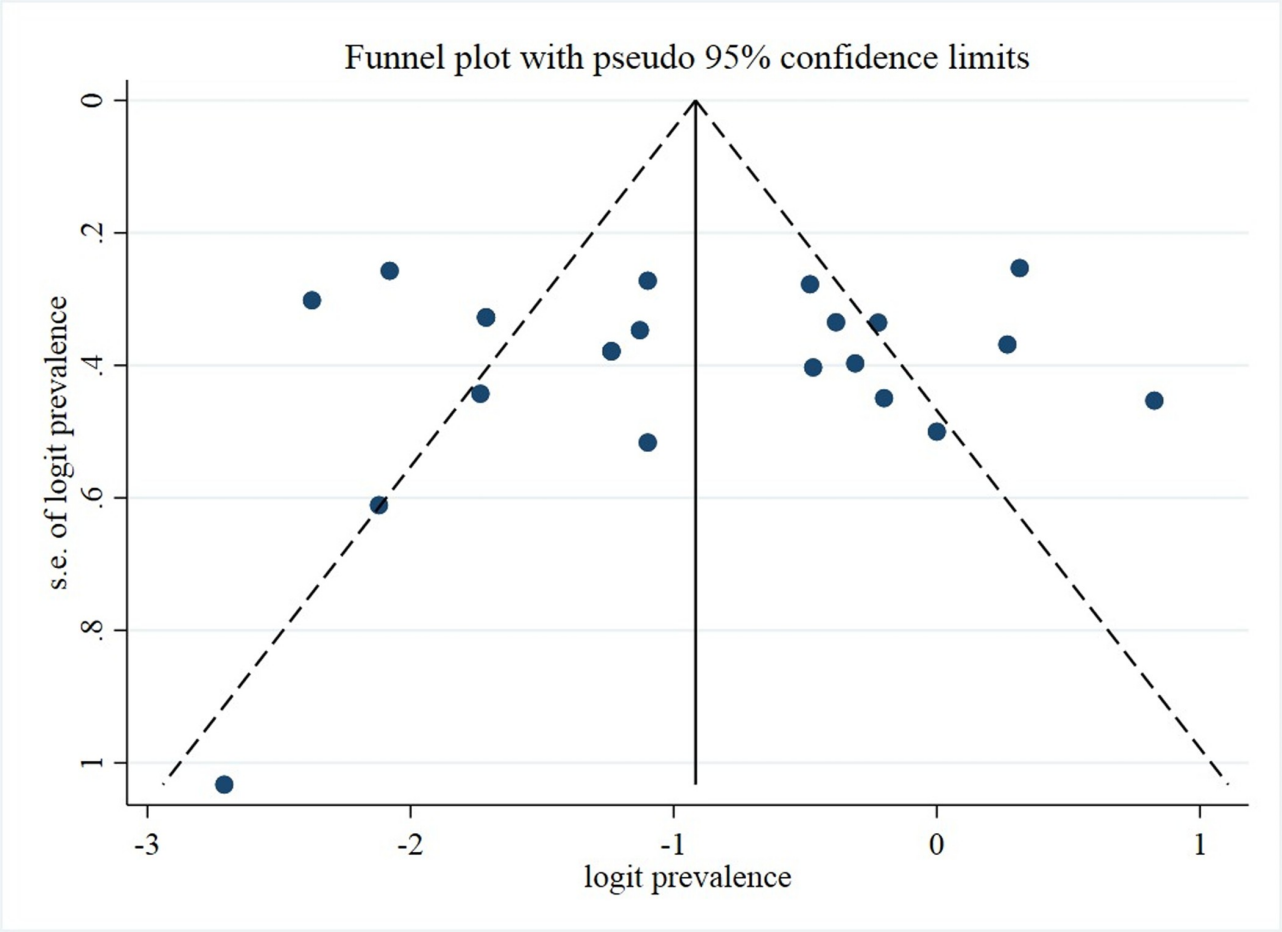
The funnel plot showing no evidence of publication bias, statistically supported by Egger’s regression test.

The pooled prevalence of olfactory among patients with MS was 27.2% (95% CI: [19.7%, 35.4%]) (*Fig 3*) with a high level of heterogeneity (I^2^ = 87.4%; p<0.001) The prevalence estimates ranged from 0% observed in Austria population to 69.6% for Italy. The funnel plot (*Fig 4*) showed no evidence of publication bias, which was statistically supported by Egger’s regression test (Bias = 0.099; p = 0.964) (S2 Table).

### Subgroup analysis

The results of subgroup analysis were shown in Table 2 by sample size, publication year, EDSS and disease duration. The pooled prevalence of olfactory was higher in studies with a mean EDSS more than 3 compared to those EDSS lower than 3 (32.6% vs. 15.9%, p = 0.048). However, prevalence of olfactory was not significantly different in terms of sample size (15% vs. 22.6%, p = 0.246), publication year (22.3% vs. 30.2%, p = 0.373) and disease duration (36.1% vs. 18.7%, p = 0.059).

**Table 2:** Subgroup analysis of pooled prevalence of olfactory.

Subgroup by		No. of studies	Total sample size	Pooled prevalence (95% CI)	Heterogeneity I^2^	p-value
Sample size	≤ 50	16	470	15% (5.7%-29.8%)	81.47	0.246
	> 50	7	629	22.6% (11.7%-35.6%)	92.17	
publication year	≤ 2010	9	399	22.3% (16.3%-28.9%)	50.97	0.373
	> 2010	14	700	30.2% (18.3%-43.4%)	91.77	
EDSS	≤ 3	8	486	15.9% (8%-25.6%)	82.94	0.048
	> 3	5	143	32.6% (19.4%-47.3%)	64.89	
Disease duration	≤ 10	7	195	36.1% (25.8%-47.1%)	56.65	0.059
	> 10	7	480	18.7% (12%-26.4%)	72.75	

### Publication bias

**Fig 5:**
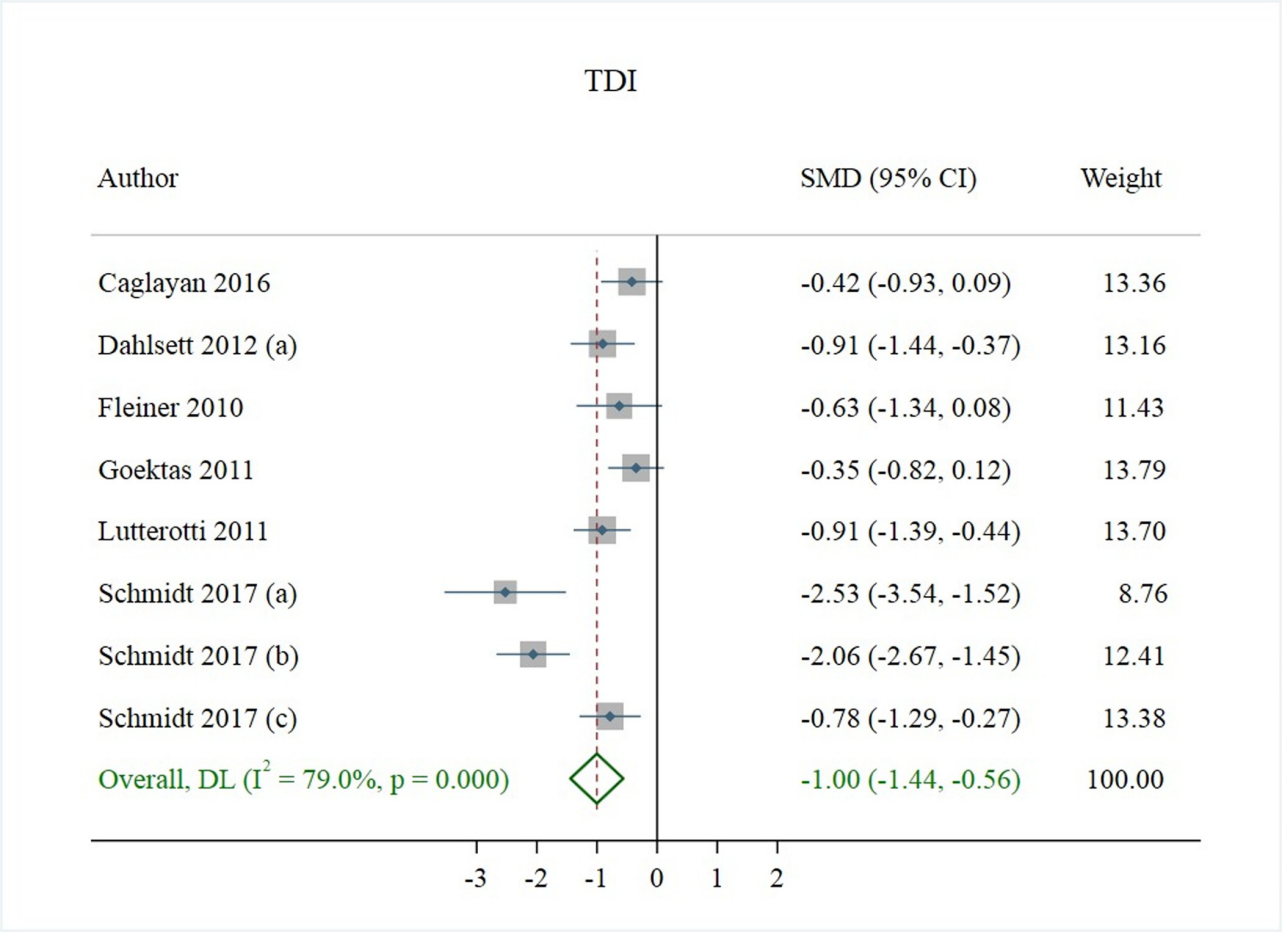
Studies reporting TDI, threshold, discrimination, and identification.

Eight studies reported TDI, Threshold, Discrimination and Identification (220 controls and 240 cases) (*Fig 5*). The overall TDI score in MS patients was lower than that in the control group (Standard Mean Difference (SMD) = -1.00; 95% CI: [-1.44, -0.56]). Also, overall level of Threshold (SMD = -0.47; 95% CI: [-0.75, -0.19]), Discrimination (SMD = -0.53; 95% CI: [-0.96, -0.10]) and Identification (SMD = -1.02; 95% CI: [-1.36, -0.68]) were lower in MS compared with control, respectively (S1–S3 Figs). Between study heterogeneity was observed in all 4 indices, however we did not find any evidence of publication bias Table 3 and there was no need for additional studies.

**Table 3:** Level of heterogeneity and publication bias amongst the included studies.

	Heterogeneity			Publication bias	
	Cochran’s Q	I2	p	Egger’s value	p
TDI	33.33	79%	<0.001	-6.522	0.076
Threshold	15.35	54.4%	0.032	-0.733	0.844
Discrimination	34.30	79.6%	<0.001	-5.934	0.177
Identification	20.10	65.2%	0.005	-4.919	0.140

## Discussion

### Overview

Olfactory dysfunctions are reported to have strong correlations with MS. Not only do MS patients show higher levels of olfactory impairment but they also forfeit this ability as their disease progress, which might be due to a lot of underlying factors such as extensive demyelination, accumulating plaque burden, and increasing cognitive impairments [1, 36, 41]. That is why the idea of using olfactory screening tests as a diagnostic and prognostic marker is capturing more and more interest every day [32, 33, 35]. To our knowledge, this is the first systematic review and meta-analysis on the prevalence of olfactory dysfunction in MS patients. Our results showed that the pooled prevalence of olfactory dysfunction among MS patients was significantly higher than the general population. Aside from general olfactory dysfunction, eight of the included studies went into detail and categorized the dysfunction as TDI score, including the Threshold, Discrimination, and Identification scores. The overall TDI score in MS patients was also lower than that in the control group.

### Overall prevalence of olfactory impairment in MS—general considerations

Our results showed that the pooled prevalence of olfactory dysfunction among MS patients was 27.2%. The highest pooled prevalence of olfactory dysfunction among MS patients was seen in Italy with a rate of 69.6%, and the lowest pooled prevalence was observed in Austria at 0.00%. Among the included studies, a study conducted in 2012 with a sample size of 153 reported that 11% of MS patients had olfactory dysfunction. This article is unique as it is one of the few studies that reported olfactory dysfunction in the control group as well, at 3%, which is significantly less than that of the MS group [31]. Moreover, a 2020-published Austrian study, Gabriel Bste et al. [27] evaluated 260 MS patients and found that 27.3% had hyposmia which is much higher than the general population. This study also reported 110 MS patients (42%) were smokers which might be used for future research into the presumably confounding association between smoking and olfactory dysfunction among MS patients. However, one of the highest rates of olfactory dysfunction among MS patients was reported by F.A. Schmidt et al. [18], who examined 64 MS patients in 2017 and revealed 57.8% had olfactory dysfunction. It is notable to mention that this was one of the few studies where the Threshold Discrimination Identification (TDI) test was performed as the screening tool, which makes it necessary to interpret the findings with caution. Olfactory dysfunction is associated with other diseases especially those affecting the CNS as well. A review article by Shin et al. [42] suggested that aside from MS, neuromyelitis optica, and systemic lupus erythematosus are related to olfactory disorders. Their study also claims that inflammation in the olfactory bulb in animal models results in olfactory disturbances. Data supports the concept that acute, self-limited inflammatory response mediates repair signaling through the NF-κB pathway and contributes to neuro-regeneration in the Olfactory Epithelium (OE) [43]. However, as the inflammation sores, it leads to a disruption in the cell-cycle regulation of the OE [44, 45]. Such disruptions eventually lead to degeneration of olfactory receptors and its related neural signaling pathways [46]. Moreover, the same pathophysiology is thought to contribute to the ever-growing report of olfactory dysfunction among COVID-19 patients [5]. As the inflammation heightens, the release of pro-inflammatory cytokines interferes with normal olfactory neural signaling pathways, leading to altered states of olfaction [47, 48]. Inflammation seems like the pivotal concept that bridges CND inflammatory diseases and COVID-19 with olfactory dysfunction, since in both cases the NF-κB pathway signaling seems to play a crucial role [30]. There is accumulating evidence on the role of this signaling pathway in exacerbated inflammation, the development of MS, MS-related sensory-neural disturbances, and COVID-19-driven olfactory dysfunction [49–51]. Overall, not only do the findings demonstrate that screening for olfactory dysfunction can help diagnose susceptible individuals sooner, but it is also of potential benefit to predict MS patients’ disease progression.

### Papers heterogeneity and possible role of the different applied diagnostic tools

Our results showed a substantial amount of heterogeneity. To get a clearer view of the reasons contributing to the heterogeneity, it is noteworthy to mention there are multiple tests to assess one’s ability of olfaction, such tests include the University of Pennsylvania Smell Identification Test (UPSIT), the Sniffin’ Sticks-Test, Odor Stick Identification Test for the Japanese (OSIT-J), Olfactory Evoked Responses Potentials (OERP), etc. Nevertheless, the golden standard of diagnosing olfactory dysfunction is the Toyota and Takagi (T&T) Olfactometer. The test utilizes its own specific kit and shall be conducted in a well-ventilated and electrically-shielded room [28]. Due to the test’s strict settings, other methods are often preferred and different studies have applied different tests based on their methodology and protocols [29, 31]. Altogether, these factors may have significantly contributed to the heterogeneity observed in present studies.

### Subgroup-analysis and possible pathological substrates

So far, existing data suggest that MS patients are at an elevated risk for experiencing olfactory dysfunction. Furthermore, our subgroup analysis showed that the pooled prevalence of olfactory dysfunction in studies in which mean EDSS was more than 3 was higher compared to other studies. recent research suggests that the intersection of persistent inflammation within the CNS, demyelination of olfactory bulbs, and the burden of plaque in brain areas associated with the olfactory system contribute to the disturbances seen in the olfaction of MS patients [1, 17]. Consequently, pro-inflammatory cytokines which are abundantly found in the CNS of MS patients have been shown to be inversely correlated with olfactory function [14, 26]. Besides, olfactory dysfunction is recognized across an ever-broadening spectrum of demyelinating conditions including MS. Demyelination and MS-plaque formation within the olfactory-related CNS regions are thought to disturb normal olfaction in the same way as it affects other sensory pathways [8, 13]. Another aspect of MS pathophysiology that becomes more apparent as the disease progresses is the cognitive impairment that the affected individuals experience. Cognitive function decline can play a major part in olfactory dysfunction as both olfactory and certain cognitive functions are controlled via the orbitofrontal cortex and that higher levels of cognitive impairment is associated with more severe manifestations of olfactory dysfunction [52]., unlike previous studies, our results did not show a correlation between olfactory dysfunction and disease duration [28], which might be due to the fact that our analysis reported a high level of heterogeneity. Overall, different aspects of MS pathophysiology seem to be working like building blocks for an altered, disturbed, and dysfunctional olfaction in affected patients.

### Strengths and limitations

Our study has some strengths. First, it is the first systematic review evaluating the prevalence of olfactory dysfunction among MS patients. Second, not only did we assess the prevalence of olfactory dysfunction in MS patients but we also estimated its pooled prevalence based on different disease duration and EDSS scores. However, we had some limitations, too. First, whether or not an association exist between the type of medication patients receive and the chance of olfactory dysfunction was not assessed. Second, although previous studies suggest individuals with primary progressive multiple sclerosis (PPMS) are at higher risk for developing hyposmia compared to relapsing remitting multiple sclerosis (RRMS) [18], independent of other disease severity measures, we were unable to conduct such a subgroup analysis since a considerable number of the studies did not clarify the subtype of the disease. If there had been the possibility to conduct the sub-group analysis, we speculate results in line with previous research would have been generated, suggesting that PPMS pathophysiology may uniquely affect the olfactory brain regions more than RRMS [18].

## Conclusion

The results of this systematic review show that the prevalence of olfactory dysfunction in MS patients is significantly higher than the general population. Also, not only is the overall collective TDI score in MS patients lower than that in the control group, but the level of Threshold, Discrimination, and Identification per se are lower in MS compared with control as well. It also provides us with insight into the importance of routine and systemic checkups in MS patients in an effort to prevent the progression of severe comorbidities.

## Supporting information

S1 Checklist PRISMA 2020 checklist. (DOCX)

S1 Table Quality assessment of the included studies using the JBI checklist. (DOCX)

S2 Table Minimal anonymized data used to conduct the meta-ananlysis. (XLSX)

S1 Fig Quality assessment of the included studies using the JBI checklist. (JPEG)

S2 Fig Overall level of threshold. (JPEG)

S3 Fig Overall level of discrimination. (JPEG)

S4 Fig Overall level of identification. (JPEG)
