# Supplementary figures and images for: Olfactory dysfunction in patients with multiple sclerosis; A systematic review and meta-analysis
Source: PLoS One. 2022 Apr 19;17(4):e0266492. doi: 10.1371/journal.pone.0266492 (PMC9017946; doi:10.1371/journal.pone.0266492)

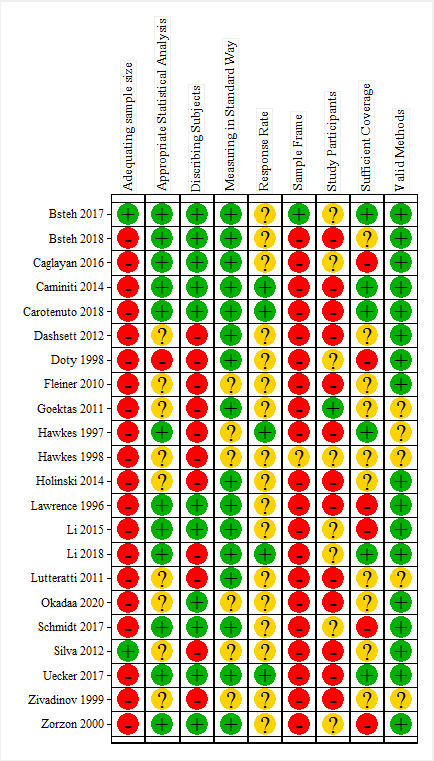

Supplement: S1 Fig — (JPEG) [file pone.0266492.s004.jpeg]

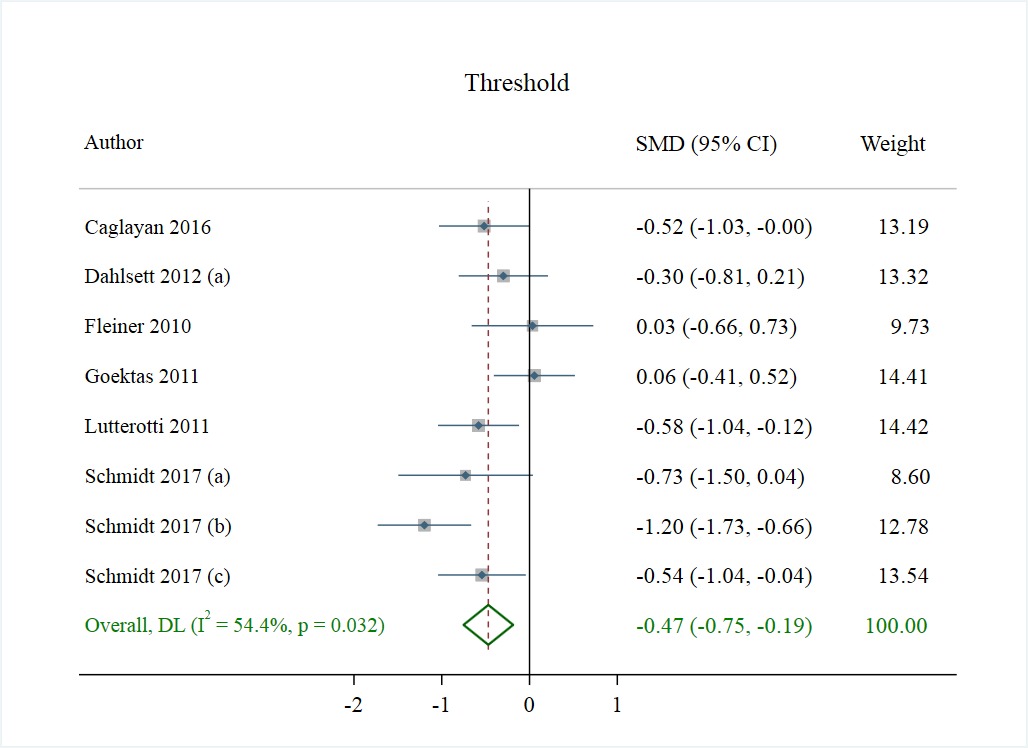

Supplement: S2 Fig — (JPEG) [file pone.0266492.s005.jpeg]

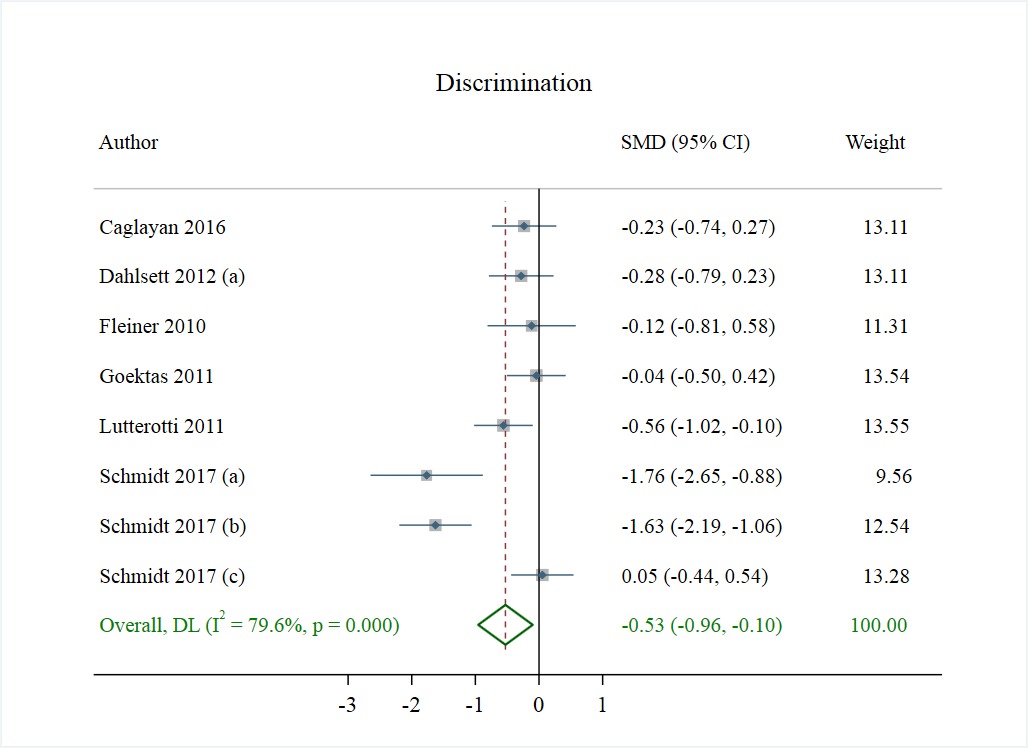

Supplement: S3 Fig — (JPEG) [file pone.0266492.s006.jpeg]

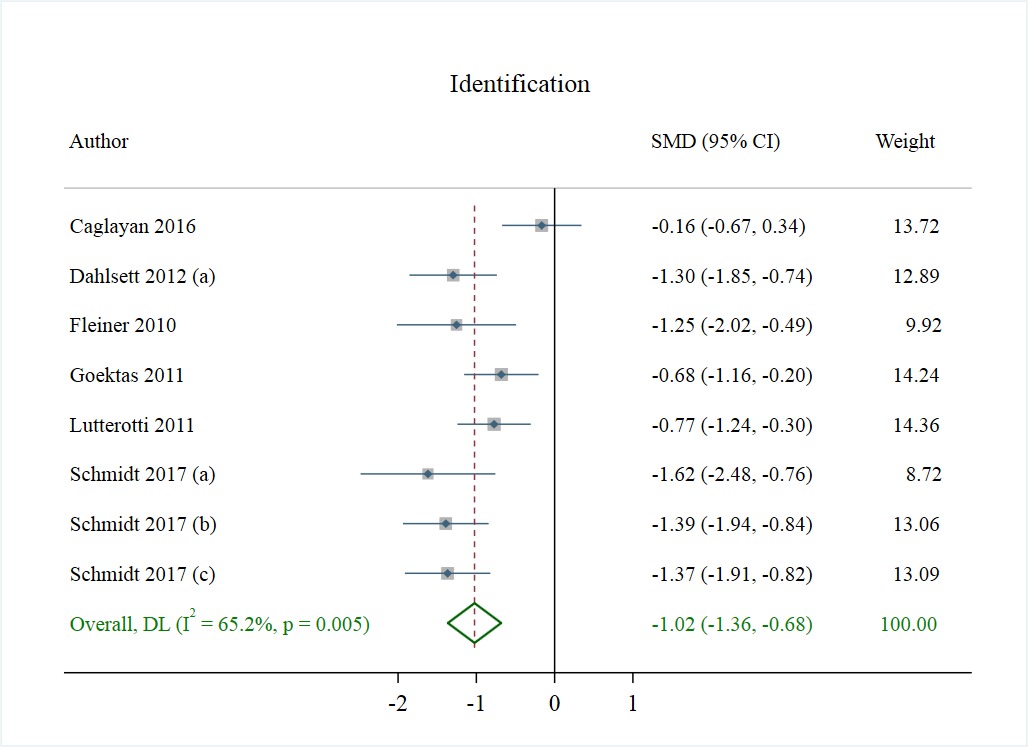

Supplement: S4 Fig — (JPEG) [file pone.0266492.s007.jpeg]
